# Recurrent *Plasmodium vivax* Cases of Both Short and Long Latency Increased with Transmission Intensity and Were Distributed Year-Round in the Most Affected Municipalities of the RACCN, Nicaragua, 2013–2018

**DOI:** 10.3390/ijerph19106195

**Published:** 2022-05-19

**Authors:** Aida M. Soto, Lilia González-Cerón, Frida Santillán-Valenzuela, María E. Parrales, Alberto Montoya

**Affiliations:** 1 Centre for Research and Health Studies, National Autonomous University of Nicaragua, Managua 11095, Nicaragua; aidasoto40@gmail.com; 2 Regional Centre of Research in Public Health, National Institute for Public Health, Tapachula 30700, Chiapas, Mexico; fsantill@insp.mx; 3 Malaria Component, Ministry of Health, Managua 11165, Nicaragua; dh62-mga@minsa.gob.ni; 4 Parasitology Department, National Centre for Diagnosis and Reference, Ministry of Health, Managua 11165, Nicaragua; parasitologia@minsa.gob.ni

**Keywords:** *Plasmodium vivax*, recurrent cases, relapse, latency, transmission intensity, malaria cases, secondary case report, Nicaragua

## Abstract

The characteristics of *P. vivax* recurrent episodes were examined using a centralized secondary source of malaria records in Nicaragua and in the two most affected municipalities in the RACCN. The study of 36,787 malaria cases due to *P. vivax* or *P. falciparum* revealed that, nationwide, 3624 patients had at least one recurrent infection. This was achieved by matching names, gender, age, community/municipality, ethnicity, etc. *P. vivax* was responsible for 88% of recurrent infections of 25–450 days of latency (51.9% were women and 48.1% were men), and these were assumed to be relapse episodes. Of them, 88.2% and 4.4% occurred in the municipalities of Puerto Cabezas and Rosita, respectively. The proportion of *P. vivax* patients having presumed relapse episodes rose with elevated transmission rates in both municipalities, reaching 7% in Rosita (2017) and 14.5% in Puerto Cabezas (2018). In both areas, relapse episodes were evident over time and were characterized by the production of a continuous stippling pattern with a slope evolving from one transmission peak to the next. During the dry season, short-latency relapse episodes were more robust, while long-latency ones increased just before the *P. vivax* transmission season began, with a high proportion of long-latency relapses during this period. The abundance of recurrent *P. vivax* infections, the wide range of relapse latency lengths, and temporal distribution tended to favor year-round transmission. It is necessary to evaluate compliance with and the effectiveness of primaquine treatment and contemplate the use of an alternative drug, among other actions.

## 1. Introduction

Malaria is a parasitic disease causing a public health problem in tropical and subtropical areas globally [1]. *Plasmodium falciparum* and *Plasmodium vivax* are the main species causing human malaria and are transmitted by *Anopheles* species [1]. The transmission of *P. vivax* is difficult to interrupt, largely because this species generates relapse episodes in patients after the primary blood infection is cured. Under certain circumstances, the dormant form of *P. vivax* (hypnozoites) develops in the liver and can be activated shortly after the infection or over a year later. Therefore, patients might suffer recurrent blood infections due to hypnozoites. The pattern of *P. vivax* relapse episodes varies [2], with about 8–80% of patients having at least one relapse episode, depending on the geographic region [3]. Regarding the latency of recurrent infections, tropical strains are expected to favor short incubation of the primary infection and several early relapse episodes, while strains from temperate and subtropical areas seem to produce hypnozoites of long or mixed latency [4].

Primaquine (PQ) has been used worldwide to treat hepatic hypnozoites since 1960 [3]. In the Americas, a total dose of 3.5 mg of PQ per kg of body weight administered within 14 days is the most effective scheme, reducing to 10% the occurrence of relapse in patients [5]. In El Salvador, without PQ treatment, 46–68% of patients developed recurrent *P. vivax* episodes by a 9-month follow-up. Contrarily, in patients that received a 5-day PQ treatment, the proportion of recurrent infections diminished to 28% [6]. An examination of the malaria records in El Salvador showed that the first recurrent *P. vivax* infection had a latency after the primary infection of 8–52 weeks (56–364 days) [7]. Similarly, in Mexico, the predominant latency period of the first relapse episode was between 31 and 352 days [8,9]. In the Mesoamerican region, a pattern of relapse episodes was detected, in which the first infection might be followed by one or various episodes with shorter latency [10].

*Plasmodium vivax* was responsible for ∼75% of the 596,200 confirmed malaria cases in the Americas in 2020, while ∼25% were produced by *Plasmodium falciparum* [11]. Malaria cases and deaths decreased by 20% in the Americas from 2010 to 2020 [11]. However, *P. vivax* has played a substantial role in the stagnation of malaria reduction efforts as of 2015 [12]. During 2015–2020, there was a drastic rise in malaria cases in the Bolivarian Republic of Venezuela, Panama, and Nicaragua, along with outbreaks in Costa Rica, the Dominican Republic, and Ecuador [11]. In Nicaragua, malaria cases declined gradually during the period of 2000–2010, reaching a total reduction of over 90% in incidence (from 23,281 cases in 2000 to 896 in 2010). The number of malaria cases fluctuated slightly in the following years (2011–2014), from 1171 to 1564 per year, and then continuously increased from 2886 in 2015 to 25,530 in 2020 [11,13], returning to the high level of transmission found before the year 2000. At present, Nicaragua reports the greatest number of malaria cases in Central America. Although *P. falciparum* and *P. vivax* coexist in Nicaragua, the latter caused more than 80% of malaria cases in 2019 and over 52% in 2020 [11]. At present, a 7-day treatment with 0.5 mg/kg/day is employed in different countries of the Americas, including Nicaragua, to eliminate *P. vivax* infections [13].

The current contribution is an observational and retrospective analysis of recurrent *P. vivax* infections in Nicaragua from 2013 to 2018. The proportion, periodicity, temporality, and parasitemia of recurrent *P. vivax* episodes were evaluated at a national level and in the two municipalities with the highest malaria transmission in the RACCN, based on a centralized secondary source of malaria records compiled by the Ministry of Health. Because *P. vivax* coexists with *P. falciparum* in Nicaragua, an additional analysis was executed to make comparisons.

## 2. Materials and Methods

### 2.1. Description of the Study Area

Nicaragua is a multi-ethnic and multicultural country located in the middle of the Central American isthmus. In 2020, the population was estimated at 6,518,478 [14] inhabitants, 51% of which were female and 49% male. Of the economically active population, 74% were under 40 years of age. The country encompasses 120,339.2 km^2^, divided into 15 departments and 2 autonomous regions, with a total of 153 municipalities.

Two of the municipalities in the North Caribbean Coast Autonomous Region (RACCN) most affected by malaria are Puerto Cabezas and Rosita (Figure S1). They have a very humid (subtropical) climate and an annual rainfall of between 1900 and 3290 mm, and the rainy season takes place from May to November. Puerto Cabezas has a multi-ethnic population of 111,803 inhabitants (predominantly Miskitos) and a territorial extension of 5985 km^2^ on the Caribbean coast. Approximately 95% of the people are under 49 years old. The economy is driven mainly by fishing, forest extraction, subsistence agriculture, animal husbandry, and informal trade. The majority of the population lives in flood zones. The urban area of the city of Puerto Cabezas is divided into 26 wards, while the rural areas of the municipality consist of 99 localities [15]. Rosita has a territory of 2205 km^2^ with 36,309 inhabitants, comprised mostly of mestizos. There are also other ethnic groups, such as the Miskitos, Magnyas, and Creoles. The principal economic activity is based on agriculture and animal husbandry. The majority of the people live in the rural areas, consisting of 80 localities. The urban area is divided into 11 wards. Malaria diagnosis is routinely confirmed by microscopy of the thick blood smear in lab facilities or by applying rapid diagnostic tests (RDTs) in inaccessible and remote areas [16]. In Nicaragua, *Anopheles albimanus* and *An. pseudopunctipennis* are the main vectors involved in the transmission of malaria. *An. albimanus* is the principal species in the rainy season throughout the country, with few exceptions. It has greater populations in the municipalities of higher transmission [17]. In contrast, *An. pseudopunctipennis* prevails in the dry season, especially in the municipality of Chinandega, because there is an abundance of hatcheries created by the conditions of sugarcane cultivation [18]. 

This study was approved by the Institutional Committee for Ethics Review (CIRE) at the Ministry of Health in Nicaragua (NIC-MINSA/CNDR CIRE-08/08/16-071). After using the patients’ names and surnames to seek evidence of recurrent infections, the identity of each patient was protected, being encrypted with a unique code before the analysis of recurrent infections was carried out.

### 2.2. Data Cleaning and Analysis

The secondary source of malaria cases was compiled on spreadsheets by the Malaria Component at the central level of the Ministry of Health. The records from 2013 to 2018 were reviewed for consistency of information by examining the first and middle name(s), surname(s), age, gender, ethnicity, locality and municipality of origin, and date of blood sample collection for parasitological diagnosis. Additional data were carefully checked, including the medication and days of treatment (the dates for starting and ending treatment), whether the delivery of the treatment was supervised “in mouth”, the date of the onset of symptoms prior to taking the blood sample for diagnosis, and the possible existence of parasitemia (by performing parasite counts). The total number of cases per malaria species was counted for the entire period and per year by utilizing the corrected databases on Excel sheets.

The analysis of data was divided into two stages. Firstly, a search was conducted to find patients with at least two malaria cases from 2013 to 2018. Accordingly, malaria records were examined for each three-year period: 2013–2015, 2014–2016, 2015–2017, and 2016–2018. Two investigators manually filtered the data, matching the identity of each patient suspected to have two or more infections. For this purpose, four columns were prepared, with the patients’ first name, middle name, first surname, and second surname. Other parameters, such as age, gender, ethnicity, locality, and municipality of the patients, were taken into account to confirm a match. A list of patients with more than one confirmed malaria case (regardless of parasite species) was compiled in an “R-database”, and the full name of each patient was encrypted by using a unique code. Secondly, the recurrent (R) malaria cases were scrutinized. A patient with a recurrent infection was defined as one with two or more malaria attacks caused by the same malaria species (perhaps mixed with an infection from the other species). Excluded from the analysis were 178 patients either duplicated or with a latency <7 days (*n* = 356 cases). Latency was expressed as the time interval in days between two consecutive infections (the primary infection and first recurrent episode (R1), R1 and R2, and so on).

In Nicaragua, because of historical coexistence of *P. vivax* and *P. falciparum* [11,19], frequencies in the recurrent cases were contrasted. If the primary infection was produced by *P. vivax* and the following infection by the homologous species or by *P. falciparum*, the second one was herein defined as a reinfection. The proportion of recurrent cases triggered by *P. vivax* and *P. falciparum* was established. Short latency was considered as probable recrudescence: 7–24 days for *P. vivax* [5] and 7–60 days for *P. falciparum* [20,21].

Calculations were performed with Microsoft Excel, and the statistical analysis (at 95% confidence) was carried out on Stata v14.2 (StataCorp LLC, College Station, TX, USA). The variables under evaluation had no normal distribution, and non-parametric statistics were used. Median and 25–75% interquartile range (IQR) were calculated for numeric variables, and the Mann–Whitney U test or the Kruskal–Wallis test of ranks was used to compare two or more independent groups, respectively. Spearman coefficient evaluated the level of correlation between two independent numeric variables. Chi-square (*χ*^2^) test was used to determine differences in the frequency of two or more independent groups.

## 3. Results

### 3.1. Malaria Cases per Species per Year at a National Level

The secondary database of malaria records revealed 36,787 cases from 2013 to 2018, of which, *P. vivax* was responsible for 86.8%. The annual number of malaria cases caused by *P. vivax*, *P. falciparum*, or mixed *Plasmodium* species is shown in Table 1. Annually, *P. vivax* cases ranged from 79.5 to 91.4%, and their prevalence in mixed infections (with *P. falciparum*) varied from 0.1 to 0.3%.

### 3.2. Patients with Recurrent P. vivax Infections Nationwide

**Figure 1:**
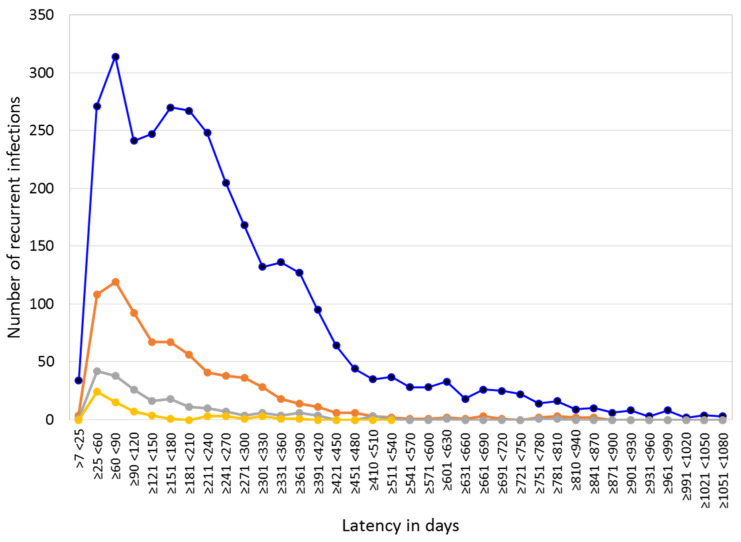
Number of patients in Nicaragua with recurrent *P. vivax* infections, categorized by latency, from 2013 to 2018. Interval between the primary infection (PI) and the first recurrence (R1) (in blue; median = 207/IQR, 113–331), between R1 and R2 (in orange; median = 109/IQR, 62–200), between R2 and R3 (in grey; median = 71/IQR, 54–132), and between R3 and R4 (in yellow; median = 64/IQR, 59–142). The latency of recurrent cases decreased as the number of R episodes increased (*χ*^2^ (3) = 136.2, *p* < 0.001).

A total of 3624 patients in the records had at least one recurrent (R) *Plasmodium* infection (by one of the two species or mixed infection) with a latency ≥7 days. Of these patients, 88.2% corresponded to a first recurrence (R1) with *P. vivax* (3196 patients, with an accumulated 7324 *P. vivax* infections or 13.2% of total *P. vivax* cases recorded). Recurrent infections were grouped by the latency between the primary infection (PI) and R1, between R1 and R2, and so on (Figure 1). Of the total recurrence of *P. vivax* episodes, 88% corresponded to R1 with a latency of 25–450 days (51.9% corresponded to women and 48.1% to men); this range of *P. vivax* latency to relapse has been suggested previously for the region [5,7]. In this group, there was a slight difference in age by gender: 22 years for women (IQR 15–34) and 21 years for men (IQR 14–32; Mann–Whitney, z = 3.009, *p* = 0.002). Aside from this, in relation to the remaining recurrent infections of longer latency, no differences in age by gender were detected (*n* = 375, z = 0.16, *p* = 0.86). No significant differences were detected between latency of recurrent cases and age or gender (*χ*^2^(5) = 8.48; *p* = 0.131) (Figure S2).

Considering the R1 latency of 25–450 days, the median was 186 days (IQR 105–275). The interval between *P. vivax* R1 and R2 episodes was shorter than that between the primary infection and R1 and so on (*χ*^2^ (3) = 136.2, *p* = 0.0001; Figure 1). The latency of R1 in patients with *P. vivax* recurrent infections was portrayed according to age and gender, and no significant difference was detected (*χ*^2^(5) = 8.48; *p* = 0.131) (Figure S2). In relation to PQ administration, 88.6% of those patients were annotated to receive the 7-day treatment, and all doses were recorded as supervised “in mouth”. Of the other 11.4% of patients, no information existed for 9%, while the medication was taken over a period of 4–34 days (excluding 7 days) for 2.4%.

There were 34 cases of recurrent *P. vivax* infection with a latency of 7–24 days (detected in both genders and for different ages; z = −0.462, *p* = 0.643). Of them, one patient had a total of three and another four *P. vivax* episodes. The recurrence of *P. falciparum* infections also took place in both genders and all ages (z = −0.307, *p* = 0.758) (Figure S3).

Of the total number of patients with recurrent *P. vivax* infections (latency of 25–450 days), 88.2% were identified in the municipality of Puerto Cabezas, 4.4% in Rosita, and 3.1% in Waspam in the RACCN (Figure S1). The rest were distributed among the remaining municipalities.

### 3.3. Proportion and Latency of P. vivax Recurrent Cases in Municipalities: Puerto Cabezas and Rosita, RACCN (2013–2018)

**Figure 2:**
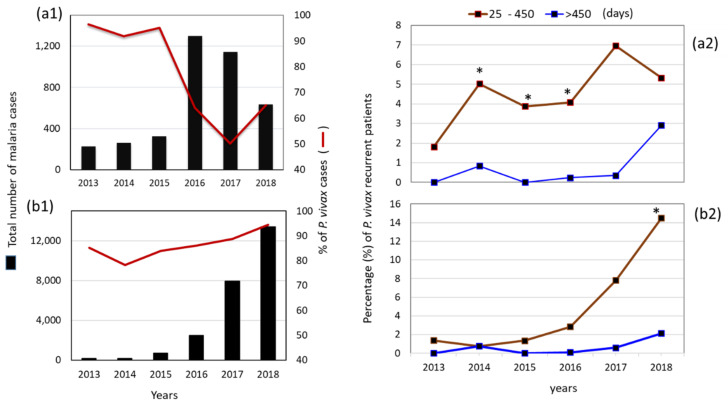
*P. vivax* cases per year and the quantity of patients with recurrent *P. vivax* infection (R1) in two municipalities of RACCN, Nicaragua, from 2013 to 2018: (**a**) Rosita, (**b**) Puerto Cabezas. In **a1**,**b1**, the number of total cases per municipality and proportion of *P. vivax* cases are indicated. Cases per year: Puerto Cabezas (2013 = 145 cases, 2014 = 133, 2015 = 588, 2016 = 2117, 2017 = 7049, 2018 = 12,683); Rosita (2013 = 221 cases, 2014 = 239, 2015 = 310, 2016 = 834, 2017 = 575, 2018 = 413). In (**a2**,**b2**, the *P. vivax* recurrent cases are represented as a percentage of the total number of *P. vivax* patients. Recurrence is illustrated according to latency (between the primary and first recurrent (R1) blood infection): from 25 to 450 days (in brown, the highest number of patients with recurrent infections) and over 450 days (in blue). For years 2014–2016, the proportion of *P. vivax* recurrent cases was higher in Rosita than in Puerto Cabezas (2014: *χ*^2^ = 4.6, *p* = 0.031; 2015: *χ*^2^ = 5.9, *p* = 0.015; 2016: *χ*^2^ = 67.5, *p* < 0.0001), and, for 2018, the percentage was higher * in Puerto Cabezas than in Rosita (*χ*^2^ = 26.1, *p* < 0.0001).

The municipalities of Puerto Cabezas and Rosita each had a distinct number of cases and proportion of *P. vivax* to *P. falciparum* infections during 2013–2018 (Figure 2a1,b1) and were selected for a more detailed analysis. The percentage of patients with one or more recurrent *P. vivax* infections with an interval of ≥25 days before R1 is illustrated in Table 2. The nation as a whole and Puerto Cabezas had a similar pattern in the proportion of patients with one or more recurrent cases (R1–R6) (*χ*^2^ = 1.7, *p* = 0.78), while, in Rosita, >90% of patients had only one *P. vivax* recurrent episode. Contrarily, in both municipalities, patients with recurrence of *P. falciparum* tended to have only one homologous recurrent infection.

During 2013–2018, the municipality of Rosita accumulated 10.5% of total malaria cases in Nicaragua. Of these, 66.5% corresponded to *P. vivax* and 33% to *P. falciparum*, with 0.4% being mixed infections. Of the *P. vivax* cases, 82% were reported for the mestizo population, followed by 13% for the Miskitos and 4.9% for the Mayangnas. Of all malaria cases in Rosita, 90% were detected by passive surveillance, with 45.5% corresponding to women and 54.5% to men. On the other hand, 67.6% of the total number of malaria cases in Nicaragua from 2013 to 2018 was located in the municipality of Puerto Cabezas, RACCN. Of these, 91.5% were triggered by *P. vivax*, 8.1% by *P. falciparum*, and 0.3% by mixed species. Passive and active surveillance strategies detected 63% and 37% of malaria recurrent cases, respectively, and 50.7% were found in women and 49.2% in men, and 95% of patients were Miskitos (the predominant ethnic group in the region).

In Rosita, 185 patients had at least one recurrent infection by a *Plasmodium* species 25–450 days after the primary blood infection. Of such patients, 66.4% displayed a recurrence of *P. vivax*, and 8.9% of them suffered from a *P. falciparum* infection. Only three had a mixed infection (*Pv*–*Pf*), considering PI or R1. Of the 2702 patients from Puerto Cabezas with at least one recurrence of a *Plasmodium* infection that occurred 25–450 days after the primary blood infection, 91% of recurrences were produced by *P. vivax*. Additionally, 2.2% of those patients with recurrent *P. vivax* infections suffered from a *P. falciparum* infection at some point during the period under study. 

Figure 2 portrays the number of *P. vivax* cases and the proportion of recurrent versus total *P. vivax* cases categorized by year. In Rosita, during 2013–2015, *P. vivax* predominated (91–95%), and, in 2016–2018, the number of cases increased, but the proportion of *P. vivax* cases decreased to 64, 50, and 65% each year, respectively (Figure 2a1). While Puerto Cabezas had fewer malaria cases than Rosita in 2013 and 2014 (145 vs. 221 and 133 vs. 239, respectively), the number of cases in Puerto Cabezas increased to 588 in 2015 and to 12,683 by 2018. The percentage of *P. vivax* infections in Puerto Cabezas in 2015–2018 varied from 83 to 94% of total malaria cases (Figure 2b1).

Recurrence with a latency of 25–450 days ranged from 1.8 to 7% of total *P. vivax* cases in Rosita (Figure 2a2) and from 0.8 to 14.5% in Puerto Cabezas (Figure 2b2). The highest proportion of recurrences was detected in 2017 in Rosita, and it was similar to the proportion in Puerto Cabezas. In this municipality, the rate of recurrent cases increased with the number of *P. vivax* cases, especially from 2016 to 2018 (*χ*^2^ = 367.8, *p* < 0.0001), while, in Rosita, this tendency was not observed (*χ*^2^ = 6.9, *p* = 0.13).

For the entire period and for *P. vivax* affected population, the proportion of recurrent infections R1 with latency of 25–450 days was higher in Puerto Cabezas (10.8%, *n* = 22,715) than in Rosita (4.8%, *n* = 2593; *χ*^2^ = 92.5, *p* < 0.0001).

### 3.4. Temporal Distribution of First Recurrent Episodes (by the Latency) and Total P. vivax Infections in Rosita and Puerto Cabezas, RACCN

**Figure 3:**
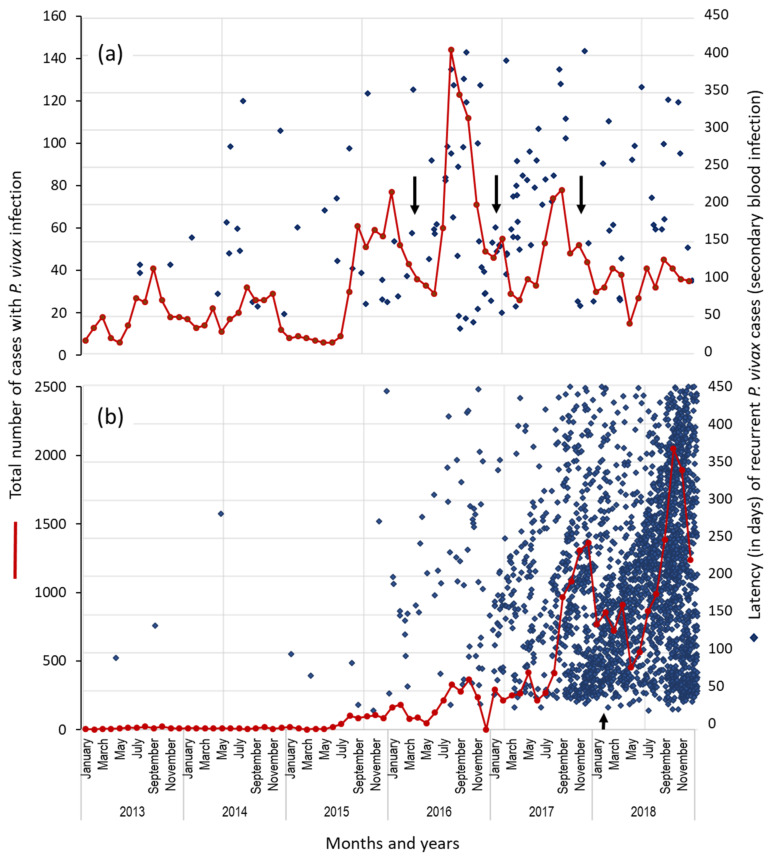
Portrayal of the temporal distribution (grouped by month) of total *P. vivax* infections from 2013 to 2018 (brown line), the first recurrent *P. vivax* infections with a latency of 25–450 days (blue diamonds). The diamonds are placed according to the interval time (in days) of the first recurrent infection after the primary infection. (**a**) Rosita; (**b**) Puerto Cabezas. R1 depicts 91.8% and 73.6% of all recurrent cases in patients affected by *P. vivax* and latency of 25–2450 in R1 in Rosita and Puerto Cabezas, respectively. Black arrows point out the extension of main transmission peaks.

The number of *P. vivax* cases and the recurrent episodes (by their latency) were accumulated on a monthly basis and plotted. It was observed that the height and width of transmission peaks varied each year and between municipalities. In Rosita, *P. vivax* cases were low during January–June 2015; after that, the transmission season began and, in August, reached a plateau and presented some fluctuations that lasted until January 2016. In the same year, in September, another sharp peak of cases was observed; then, the number of cases descended gradually to a moderate level for three months (from December 2016 to February 2017). In April 2017, the cases decreased to the lowest number (Figure 3a). Afterward, the number of cases per month was low and fluctuated over time. Regardless of the number of recurrent infections (R1), a stippling pattern was apparent, with a slope from one transmission peak to the next one, which is most evident from 2015 to 2017 (Figure 3a). In Puerto Cabezas, *P. vivax* cases increased discretely in August–December 2015, rising even more after June 2016, with a plateau that continued until June 2017. Afterwards, the cases increased rapidly, presenting a peak in December that, in the following month, decreased to a moderate level which it maintained until April 2018, after which, in May, it decreased. Again, in June, the cases increased and reached a peak in October. The cases decreased in December, the last month analyzed. In this municipality, the recurrent infections formed a stippling pattern (as found in Rosita), which normally emerged from the peaks of cases to beyond next peak. The recurrent cases of all intervals increased with transmission in the latter years. Indeed, a very crowded dotting pattern resulted for 2017–2018 when plotting only R1 cases (about 73% of all recurrent cases detected). When transmission approached its peak in November 2017, 1400 *P. vivax* cases occurred in just one month: four times more than in the previous year.

**Figure 4:**
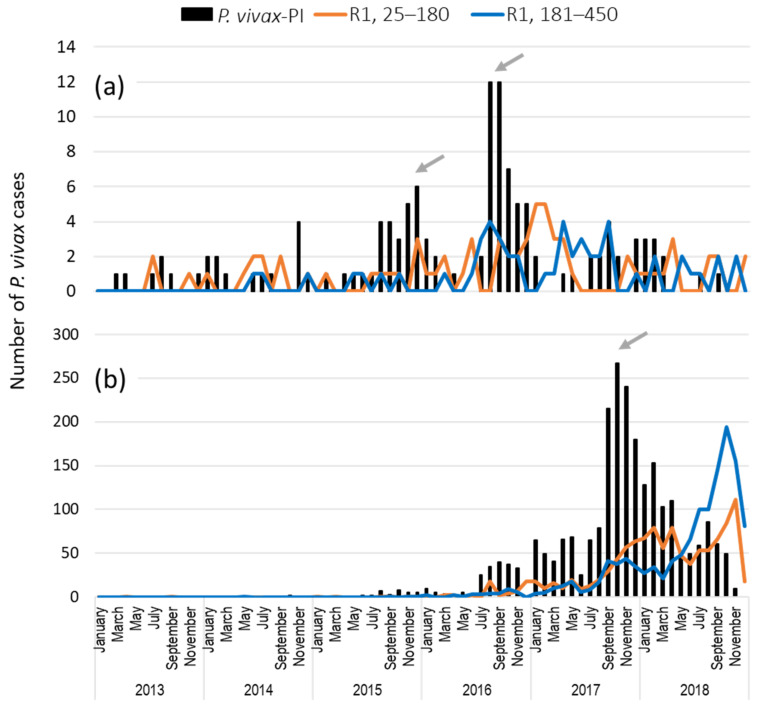
Dynamics of short- and long-latency *P. vivax* recurrent cases (R1) and for primary infections (PI, only those connected to posterior recurrent episodes) in Rosita (**a**) and Puerto Cabezas (**b**), RACCN, Nicaragua. In both municipalities, recurrent cases with short (25–180 days) or long (181–450 days) latency were present before and/or during the peak of transmission with certain alternation at some time periods. The transmission peak taking place in 2018 was not evaluated, since at least a one-year window ahead is required (2019 malaria cases). Grey arrows point out the peaks of transmission drawn in Figure 3.

Comparison of the frequencies of recurrent cases categorized as short (25–180 days) or as long (181–450 days) latency that occurred during the dry and rainy seasons, comprising the high transmission peaks in Rosita and Puerto Cabezas, was carried out (Figure 4, Table 3a,b). In both municipalities, when the high malaria transmission season took place, recurrent cases of both latency periods were found to fluctuate across time (Figure 4). In Rosita, during the rainy season of 2016 and 2017, a higher proportion of recurrent cases of long latency than of short latency was detected (Table 3a). For the same years and during the dry season, an opposite pattern was observed; a higher proportion of recurrences of short latency than of long latency was estimated. In Puerto Cabezas, during the period of the highest transmission in 2017, 52.6% of recurrent cases were of short latency, and, in the next period, this proportion increased to 65%. It was observed that moderate transmission was maintained until April the next year (2018) (Figure 3b, Table 3b). In the following period, the proportion of short versus long recurrent cases was inverted; the long-latency ones increased to 65% (Table 3b). In Rosita and Puerto Cabezas and for all periods analyzed, *P. vivax* primary cases linked to posterior recurrence were detected at different proportions, and the overall proportion was higher for Puerto Cabezas (*χ*^2^ = 150.4, *p* < 0.0001; Table 3a,b). Moreover, the higher number of those cases coincided with the period of the maximal number of *P. vivax* cases recorded (Figure 4).

No differences were detected in the distribution of days of latency within 25–450 days between R1 cases from Puerto Cabezas and Rosita (z = 0.44, *p* = 0.65) nor between genders (*χ*^2^ = 0.20, *p* = 0 0.64). Regarding age, the female group from Puerto Cabezas was older when compared to that from Rosita (Rosita: *n* = 61, median = 19/IQR 14–16 vs. Puerto Cabezas: *n* = 1265, median = 23/IQR 15–35; z = −2.479, *p* = 0.013).

### 3.5. Parasitemia (Parasites/µL) in Patients with Recurrent P. vivax Infections

From the total *P. vivax* recurrent patients with a latency of 25–450 days, 73% and 80% had records of parasite density for the primary and R1 cases, respectively. These data were registered for years 2017 and 2018 only, and the vast majority (>94%) of cases with a recurrent *P. vivax* infection and parasite density were from Puerto Cabezas, RACCN. Spearman’s rho value between parasitemia in PI or R1 infections and age was close to zero (*n* = 1986; Spearman’s rho = −0.06; *p* = 0.029). Additionally, there was a poor positive correlation between parasitemia in the *P. vivax* primary infection and the days with symptoms prior to diagnosis (*n* = 1903; Spearman’s rho = 0.209; *p* < 0.001). Moreover, parasitemia in R1 and the latency of the same had a negative and very low value for Spearman’s rho = −0.109 (*n* = 2123, *p* < 0.001).

**Figure 5:**
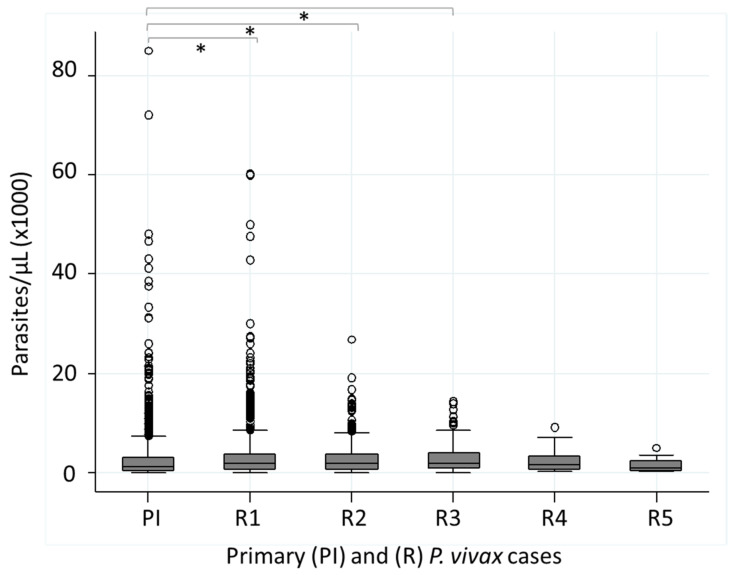
Distribution of *P. vivax* parasitemia (parasites/µL) in relation to the primary and recurrent blood infections in patients from Puerto Cabezas, RACCN, Nicaragua. Aggregated data of the primary infection (*n* = 1986) and recurrent infections, R1 (*n* = 2123), R2 (*n* = 545), R3 (*n* = 132), R4 (*n* = 37), and R5 (*n* = 17). Kruskal–Wallis: *χ*^2^ (5) = 79.7, *p* < 0.001. The comparisons with significance at 95% are indicated (*). PI (median = 1200, IQR 360–3120) versus R1 (median = 1740, IQR 660–3870); z = −7.78, *p* < 0.001. PI vs. R2 (median = 1860, IQR 720–3750; z = −5.95, *p* < 0.0001), and PI vs. R3 (median = 1875, IQR 885–3990; z = −3.76, *p* = 0.0002).

Cumulative data of parasite densities for primary and recurrent *P. vivax* infections are depicted in Figure 5. A parasite density of higher than 10,000 parasites/µL was more frequent in R1 (*n* = 2123, 19%), R2 (*n* = 545, 9.8%), and R3 (*n* = 312, 16.2%) than in PI (*n* = 37, 3.7%). Therefore, recurrent infections (R1–R3) exhibited a higher median for parasite density than the primary infections (Figure 5). Regardless of the latency, a significant difference was detected between groups (Kruskal–Wallis: *χ*^2^ (5) = 79.7, *p* < 0.001).

Furthermore, a poor correlation was detected when comparing parasite density in the primary and R1 infections (*n* = 1784, Spearman’s rho = 0.138; *p* < 0.0001), as well as between R1 and R2 infections (*n* = 495, Spearman’s rho = 0.109, *p* = 0.015); instead, blobs of dots were observed (Figure S5). At the individual level, the parasite density for PI and subsequent recurrent infections showed no pattern (some examples are provided in Figure S6).

In regard to asymptomatic parasitemia, only 34 cases were categorized for primary infections in *P. vivax* recurrent patients. Of the first recurrent infections (R1), there were 108 presumably asymptomatic cases versus 2015 symptomatic cases with parasitemia records; the former displayed a lower density of parasites in the blood than the latter (median = 960 (IQR, 360–1830) versus 1800 (IQR, 690–3960) parasites/µL (z = 5.23, *p* < 0.0001).

For Rosita, parasite density was recorded in few PI cases (*n* = 23, median 2700, IQR 701–6045) and R1 episodes (*n* = 40, median 2108, IQR 693–3730); only the parasite density of PI was higher than that from Puerto Cabezas (z = 2.74, *p* = 0.006), but no differences in R1 were estimated (z = 0.22, *p* = 0.82).

### 3.6. Comparison of Recurrent P. vivax and P. falciparum Infections (25–450 Days) in RACCN Municipalities: Puerto Cabezas and Rosita

Rosita depicted a lower rate of *P. vivax* cases (66.8%, *n* = 3881) than Puerto Cabezas (94.5%, *n* = 24,036; *χ*^2^ = 3024, *p* < 0.0001). In Rosita, from the total recurrent cases with a latency of 25–450 days (*n* = 185), 66.5% had homologous *P. vivax* infections while 8.6% experienced homologous *P. falciparum* infections. Another 24.8% were presumably reinfections: 9.7% with a *P. vivax* recurrence after primary infection of *P. falciparum* and 15.1% vice versa (Figure S6a). Of patients in Rosita with a *P. vivax* primary infection, a second episode occurred with *P. vivax* for ∼4.8%, and, in *P. falciparum* patients affected by a homologous species, recurrent cases were fewer (∼1.2%, *n* = 1288); *χ*^2^ = 30.8, *p* < 0.0001). In Rosita, for the period 2016–2018, differences in the proportion of recurrent patients were detected between *P. vivax* and *P. falciparum* (*χ*^2^ = 20.0; *p* < 0.0001); a mere subtraction of the *P. falciparum* recurrent cases estimated that at least 60% of *P. vivax* recurrent cases of latency within 25–450 days were not reinfections. 

In Puerto Cabezas, from the total number of recurrent episodes R1 (*n* = 2702), *P. vivax* recurrent patients accounted for 90.9%, while 0.4% of *P. falciparum* patients had a recurrent infection. Patients with reinfections (caused by heterologous species) accounted for 8.5%; 5.5% involved *P. falciparum* primary infections followed by *P. vivax*, and 3% involved *P. vivax* primary infections followed by *P. falciparum* (Figure S6b). In this municipality, the number of patients diagnosed with primary and recurrent infections of *P. vivax*, 10.8%, was higher than the number of patients with primary *P. vivax* and recurrent *P. falciparum* infections (0.05%, *n* = 1321) (*χ*^2^ = 2560, *p* < 0.0001). 

## 4. Discussion

The resurgence of parasitemia and malaria in a previously infected individual can be due to reinfection, recrudescence, or relapse. Following the inoculation of *P. vivax* sporozoites into a person by an *Anophelese* mosquito species, these cells eventually enter the liver and, under certain circumstances, remain dormant in the form of hypnozoites. This dormant stage of *P. vivax* may be activated weeks, months, or years later and, thus, produce a new blood infection or a relapse episode [22]. PQ is the worldwide drug of choice for treating *P. vivax* infections in order to prevent relapse episodes [3]. Adequate doses and the completion of the treatment are key elements for reducing the frequency of relapse in patients [23,24]. While malaria cases in Nicaragua were decreasing in 2006, treatment based on chloroquine (25 mg/kg administered in 3 days) and PQ (0.5 mg/kg daily given over 7 days) was implemented. Its delivery involved supervision and cooperation of volunteer personnel from the communities (Col-vols) [25]. During the period herein analyzed (2013–2018), an outbreak of *P. vivax* cases took place in Nicaragua, and *P. vivax* infections exceeded those of *P. falciparum*, ranging from 71 to 98% of the total number of infections every year [12]. 

Based on the present data, at least 13.4% of *P. vivax* cases recorded in 2013–2018 corresponded to recurrent episodes. Of the total first recurrent infections (R1), the ones occurring 25–450 days (approximately 1–15 months) after the primary infection constituted 88%, with a median interval of 186 days (105–275). Findings from Central America support the fact that recurrent episodes R1 of 25–450 days might be related to relapse episodes. In this region, the latency from the primary infection to the first relapse is variable, comprising short and long intervals [2]. In El Salvador, recurrent infections that were relapse episodes apparently happened 1 to 14 months after the primary infection [7]. From 1998 to 2008, in southern Mexico, a hypo-endemic region, 8% of *P. vivax* primary infections led to recurrent infections. For the majority of cases, the time interval between these two infections was 4–52 weeks [8], similar to that found in the municipality of Rosita. According to the current results and assuming that, in Nicaragua, recurrent cases of 25–450 days were relapse episodes, the latency is probably not dependent on gender or age as no statistical associations were detected. 

The municipalities of Puerto Cabezas and Rosita in the RACCN each displayed a distinct incidence of malaria. Puerto Cabezas was the site of 67.5% of the nationwide malaria cases, 94% of which were due to *P. vivax*. In contrast, Rosita hosted 10.5% of the nationwide malaria cases, with 66.5% due to *P. vivax*. The rate of recurrence of *P. vivax* infections in either municipality was related to *P. vivax* transmission intensity. In this sense, the highest proportion of first recurrent *P. vivax* episodes (with a latency of 25–450 days) was 14.5% and 7% in Puerto Cabezas in 2018 and in Rosita in 2017, respectively, coinciding with the high transmission intensity taking place the previous year. In Puerto Cabezas, the impact of the highest transmission peak observed in 2018, given by the number of cases, might have been reflected in the next year. In this scenario, more than 80% of patients with recurrent *P. vivax* infections seemed to receive a 7-day PQ treatment under supervision. An increase in recurrent infections in the presence of an elevated level of transmission was also observed in different geographical areas even with the use of PQ treatment, such as in Oaxaca, Mexico [26].

In Puerto Cabezas and Rosita in the RACCN, malaria transmission is seasonal and fluctuates over time. The main vector is *An. albimanus*, which is highly abundant during the rainy season [17,27] and contributes to active malaria transmission, procreating hypnozoite carriers, and preserving the genetic pool of parasites, as seems to be the case in other areas [6,8,10]. However, evidence of vector species that support the continuation of transmission during the dry season when *An. albimanus* densities are considerably reduced is required. The fact that hypnozoite carriers are highly generated during active transmission supports that recurrent cases come from the activation of hypnozoites. In this study, relapses of long latency were observed just before and in the first few months after transmission season begins, and those of short latency were more frequent 1–2 months after transmission began, across this season and until the middle of the dry season. The number of recurrent *P. vivax* infections, the wide-ranging gradient of time intervals to relapse, and the temporal distribution (in the rainy and dry seasons) guarantee year-round parasite transmission. Both short- and long-latency relapse episodes were present during the dry season. Likewise, during this season in El Salvador, at least 60% of patients with *P. vivax* symptomatic relapse episodes were present, according to estimates [10]. Short- and long-latency recurrent *P. vivax* infections at different rates might have sustained transmission over time in Nicaragua.

Based on an analysis of malaria records in Iran, about 80–90% of malaria patients were provided with a 14-day PQ treatment (1994–2001), resulting in 16–24% recurrence with a latency from 3 to 18 months, found at a peak from 9 to 12 months [28]. A recent multicenter study encompassing Afghanistan, Ethiopia, Indonesia, and Vietnam reported that patients given either a 14- or 7-day treatment scheme had 2% versus 16% chance of relapse, respectively, within 12-month follow-up [29]. In the Americas, a total dose of 3.5 mg of PQ per kg of body weight administered within 14 days is the most effective scheme, lowering to 10% the occurrence of relapse in patients [5]. In Mexico (in Chiapas), with a supervised 14-day PQ treatment and a 12-month follow-up, 15% of patients had a relapse of long interval. In the same region, 50% of patients that received single doses had one or more relapse episodes (at a wide range of time intervals) after the primary infection [9]. In Suriname, South America, 10% and 30% of recurrent *P. vivax* infections took place (before a three-month follow-up) subsequent to a 14- or 7-day treatment, respectively [30]. A study in Brazil described a higher frequency of relapse episodes within a time interval of 25–100 days [31]. Upon reviewing 23,365 recorded cases of malaria in 2009, relapse episodes were detected in 23% of the Brazilian patients, and these were mostly with a short latency of 4–13 weeks (30–90 days) [32]. A 7- to 9-day PQ treatment is recommended in this country [33], and 75% of patients reportedly complete the treatment [34].

It may not be possible to extrapolate the outcome of the 7-day treatment in South America to Nicaragua, as both areas differ in their relapse temporality [2,6,7,8,9]. A limited PQ treatment could be effective for diminishing short-term relapses while, at the same time, compromising the reduction of long-term relapses [6,22]. In southern Mexico, a 14-day treatment prevented 100% of relapses before a 6-month follow-up and 86% from 6 to 12 months [9]. Cedillos et al. [6] reported that a 5-day PQ scheme mainly prevented relapse episodes within the first 3 months. Globally, limited evidence exists on the effectiveness of PQ administered for 7 days [5], and information is not available for Mesoamerica.

Because, in *P. vivax*, clinical immunity is acquired quickly [35,36] parasite density is expected to be lower in relapse episodes compared to the primary infection, especially in the event of genetically homologous infections, which often occur in low-endemic areas [8]. In contrast, parasitemia levels recorded in patients diagnosed in Puerto Cabezas point to a higher parasite density in recurrent cases; however, these data cannot infer transmissibility as no gametocyte densities were recorded. The lack of correlation between parasite density and the latency to relapse encountered in this study was reported earlier using the St. Elizabeth strain; long latency seems not to favor the increase of parasite density [37]. In southern Mexico (as in Nicaragua), parasitemia in R1 was not strongly associated with the latency for this infection [9]. The lack or poor correlation between parasitemia and age might mirror the effect of the recent malaria outbreak in Puerto Cabezas. In the current contribution, the population of all ages was presenting symptomatic recurrent infections due to *P. vivax* parasites. Even at an individual level, the parasite density seemed to vary greatly from the first to fourth infections.

In addition, the level of parasitemia is apparently influenced by the opportune and effectiveness of the treatment. Patients without treatment can harbor a blood infection for weeks or months as a chronic infection [38,39]. Furthermore, in endemic regions, constant exposure to the parasite induces clinical immunity, meaning that asymptomatic infections could possibly exceed symptomatic ones in some areas [40]. Fewer asymptomatic cases were recorded in Nicaragua apparently with lower parasitemia than symptomatic infections. Consequently, there was probably an additional burden of undiagnosed and unrecorded asymptomatic recurrent infections. Parasitological and RDT tests are known to commonly miss a proportion of submicroscopic parasitemia [40,41].

The present study might have discovered only a portion of the recurrent infections in endemic areas of Nicaragua. There are few published molecular studies focusing on an analysis of paired *P. vivax* samples in the Mesoamerican region. Whereas 89.7% and 37% of homologous and heterologous recurrent infections, respectively, had a latency of 31–448 days after the primary infection in southern Mexico, a longer interval characterized most subsequent recurrent infections [8]. In Nicaragua, several genotypes were reported for the blood stage antigen *pvmsp1*_42_ [42] and the multidrug resistance gene *pvmdr1* [43] in samples collected during 2012–2013 when transmission was at a low level. This information is useful as a baseline to elucidate persistent genotypes and to examine recurrent *P. vivax* infections in the affected areas as part of surveillance strategies. 

Unlike that which was reported previously [44,45], no relation existed between *P. vivax* infections and recurrent *P. falciparum* infections. Given the similar rate of *P. vivax* and *P. falciparum* infections in Rosita during 2016–2018, a more similar risk of reinfection was assumed. Considering primary *P. vivax* infections that were followed by a recurrent *P. vivax* (∼4.8%) or *P. falciparum* infection for (∼1.5%), the 65% *P. vivax* relapsing episodes estimated in Rosita followed the previously reported pattern in Iran [28]. This is lower than the 76% rate of recurrent *P. vivax* infections (as a function of total relapse episodes) described in a study involving a diverse range of *P. vivax* endemic settings [46]. Since *P. vivax* predominated in Puerto Cabezas, and transmission was high from 2015–2018, reinfections should have been more numerous, as observed elsewhere in the face of elevated transmission [47]. To understand transmission dynamics, therefore, it is necessary to analyze infective bites, among other factors.

In Nicaragua, malaria is associated with extreme poverty, social inequity, and low educational level, as well as the isolation of some ethnic groups [48,49]. Changes in socioeconomic factors triggered human displacement into areas with low malaria transmission or none at all, thus, having poor antimalarial measures. This phenomenon might have contributed to the resurgence and increase of malaria transmission; a large population movement occurred within the RACCN from Waspam to Puerto Cabezas and also from Honduras to Nicaragua just before the outbreak began. These two countries share the Miskito ethnic region, the borders of which are geographically delimited by the Coco River; however, there is free flow of the “Miskito” population [46]. Other factors identified previously were the high population density living in overcrowded situations in flood zones, lack of surveillance on the border, the irregular service of the GISI network in 2012–2013, and the limited involvement of the municipalities in malaria control measures [25,49].

Regarding the task of diminishing malaria transmission in Nicaragua, the combination of routine diagnosis, treatment of infected patients, and vector control may be insufficient. Tracking recurrent *P. vivax* infections through surveillance strategies is highly recommended, as is an evaluation of the feasibility of employing a unique malaria identification technique, as previously suggested [33,50]. Since the reduction of transmission will potentially require strategies beyond supervising the completion of treatment for 70% of patients, alternatives must be considered, such as the improvement of supervised or semi-supervised treatment strategies and post-infection monitoring with a parasitology test applied monthly for up to 6 months [34].

The main problem with the PQ treatment is that it requires supervision, optimally, for 14 days. A possible alternative is tafenoquine (TQ), which can be given in a single dose of 300 mg/kg. TQ showed similar efficacy as the 14-day PQ scheme (0.5 mg/Kg weight), and no safety issues were detected [51]. TQ was recently approved by the FDA (https://www.fda.gov (accessed on 17 May 2022)) and TGA in Australia (https://www.tga.gov.au (accessed on 17 May 2022)). However, its administration is limited to people over 16 years of age [52]. It is necessary to assess the performance of TQ against long-term relapse episodes, as well as the 7-day treatment with PQ in Mesoamerica. Mass treatments with radical cures can be deployed in areas with substantial transmission to drastically decrease *P. vivax* infections [53,54].

## 5. Conclusions

The analysis of malaria cases before and during the outbreak of *P. vivax* in Nicaragua revealed that the enormous increase in recurrent *P. vivax* infections correlated with high transmission intensity. In the municipalities of Rosita and Puerto Cabezas in the RACCN region, both short- and long-latency relapse episodes (of 25–450 days) occurred throughout the year. However, short-latency (25–180 days of latency) relapse episodes were more evident during the dry season and those of long latency (181–450 days of latency) were more obvious just before malaria transmission season began and across the rainy season. The number of recurrent *P. vivax* infections, the wide range of latency to relapse lengths, and the temporal distribution of such episodes favor the continuity of transmission. Although there was a similar incidence of *P. vivax* and *P. falciparum* infections from 2016 to 2018 in Rosita, 60% of recurrent cases within 25–450 days were apparently caused by the relapse of a *P. vivax* primary infection. A higher estimate was reported in the systematic review by Commons et al. [46]. The continuation of the observational analysis of the secondary source of malaria records from the most affected areas of Nicaragua, such as Puerto Cabezas, might contribute to the understanding of transmission dynamics of recurrent cases, improve the recording of malaria cases, and direct anti-malarial actions. Parallel to vector control, it is necessary to evaluate the effectiveness and compliance of the current treatment, as well as the possible use of alternative drugs. Moreover, malaria prevention strategies and surveillance of recurrent cases must be strengthened.

## Figures and Tables

**Table 1:** Malaria records from the secondary database in Nicaragua (2013–2018).

*Plasmodium* Species	Years (Number of Cases)						Total
	2013	2014	2015	2016	2017	2018	
*P. vivax*	950	924	1880	4343	8959	14,501	31,557
*P. falciparum*	211	139	320	1104	2033	1319	5126
Mixed *Pv*–*Pf*	0	0	3	17	35	49	104
Total	1161	1063	2203	5464	11,027	15,869	36,787
% *P. vivax*	81.8	86.9	85.3	79.5	81.2	91.4	85.8
% *P. falciparum*	18.2	13.1	14.5	20.3	18.4	8.3	13.9
% *Pv*–*Pf*	0	0	0.1	0.3	0.3	0.3	0.3
NI	4	2	0	70	0	1	77

NI, the malaria species is not indicated and not included in the counts; *Pv*, *P. vivax*; *Pf*, *P. falciparum*.

**Table 2:** Percentage of patients with recurrent *P. vivax* infections (with an interval ≥25 days between the primary infection and the first recurrence) versus total patients with recurrent infections in Nicaragua and municipalities of RACCN during the period of 2013–2018.

Number of *P. vivax* Infections	Nationwide (%) *n* = 3163 Patients	RACCN, Puerto Cabezas (%) *n* = 2789 Patients	RACCN, Rosita (%) *n* = 139 Patients
2	77.2	75.3	90.7
3	16.7	17.9	7.2
4	4	4.4	2.1
5	1.2	1.3	0.7
6	0.6	0.7	-
7	0.09	0.1	-

RACCN, North Caribbean Coast Autonomous Region. The frequencies of patients with different recurrent infections between Puerto Cabezas and Rosita were different (*χ*^2^ = 14.1, *p* = 0.006), but there was no difference between data nationwide versus data for Puerto Cabezas (*χ*^2^ = 1.71, *p* = 0.78).

**Table 3:** (**a**) Comparison of *P. vivax* short and long latency recurrent cases and primary cases linked to recurrence between rainy and dry seasons in Rosita, RACCN, Nicaragua (June 2015–November 2017); (**b**) Comparison of *P. vivax* short and long latency recurrent cases and primary cases linked to recurrence between rainy and dry seasons in Puerto Cabezas, RACCN, Nicaragua (December 2016–November 2018).

**(a)**						
**Parameters**	**Season/Period**					**Statistics (Comparing All Groups)**
	**Rain**	**Dry**	**Rain**	**Dry**	**Rain**	
	**June–November 2015**	**December 2015–May 2016**	**June–November 2016**	**December 2016–May 2017**	**June–November 2017**	
Number of *P. vivax* cases	216	302	533	241	338	*χ*^2^ = 9.6, *p* = 0.046
Linked to recurrent patients (%)	8.3	3.9	7	3	3.7	
Number and latency of R1:						*χ*^2^ = 11.04, *p* = 0.026
*n*	6	9	21	28	13	
Short: 25–180 days (%)	66.6	88.8	33.3	71.4	15.3	
Long: 181–450 days (%)	33.3	11.2	66.7	28.6	84.7	
Statistics:		*χ*^2^ = 7.7, *p* = 0.005		*χ*^2^ = 16.6, *p* < 0.0001		
	*χ*^2^ = 1.1, *p* = 0.69		*χ*^2^ = 9.03, *p* = 0.007			
**(b)**						
**Parameters**	**Season/Period**					**Statistics (Comparing All Groups)**
	**Dry**	**Rain**	**Dry**	**Rain**		
	**December 2016–May 2017**	**June–November 2017**	**December 2017–May 2018**	**June–November 2018**		
Number of *P. vivax* cases	1439	4251	5062	n.d.		*χ*^2^ = 76.7, *p* < 0.0001
Linked to recurrent patients (%)	20	20	14 *	n.d.		
Number and latency of R1:						*χ*^2^ = 211.8, *p* < 0.0001
*n*	142	325	598	1111		
Short: 25–180 days (%)	64.7	52.6	65	35		
Long: 181–450 days (%)	35.3	47.4	35	65		
Statistics:	*χ*^2^ = 5.9, *p* = 0.014		*χ*^2^ = 132.2, *p* < 0.0001			
		*χ*^2^ = 10.1, *p* = 0.001				

For latency of R1, only significant differences between periods are indicated. For the period June–November 2018, the number of *P. vivax* cases linked to upcoming recurrent cases was not possible to determine (not determined = n.d.), information of recurrent cases for 2019 is not included. The number of recurrent cases was calculated for the same period as they were mostly linked to primary cases that occurred in 2017. * numbers might be underestimated as no information from 2019 was included in the analysis.

## Data Availability

Supporting data are not available.

## Supplementary Materials

The following supporting information can be downloaded at: https://www.mdpi.com/article/10.3390/ijerph19106195/s1, Figure S1: Geographic location of Nicaragua and most affected municipalities by malaria transmission.; Figure S2: The time interval (≥25 days) between the primary case (PI) and first recurrent *P. vivax* case (R1) in Nicaragua from 2013 to 2018, depicted by gender and age group; Figure S3: Distribution of patients with short-latency recurrent *P. vivax* and *P. falciparum* infections by age and gender. Figure S4: Correlation of parasitemia in consecutive *P. vivax* infections in Puerto Cabezas, Nicaragua from 2017 to 2018; Figure S5: Variation of parasite density in primary and recurrent infections at a patient level. Figure S6: Comparison of *P. vivax* and *P. falciparum* recurrent infections and reinfections in two municipalities of the RACCN, Nicaragua, 2013–2018.
