# Supplementary material for: Recurrent Plasmodium vivax Cases of Both Short and Long Latency Increased with Transmission Intensity and Were Distributed Year-Round in the Most Affected Municipalities of the RACCN, Nicaragua, 2013–2018
Source: Int J Environ Res Public Health. 2022 May 19;19(10):6195. doi: 10.3390/ijerph19106195 (PMC9142003; doi:10.3390/ijerph19106195)
Supplement: Supplementary file 1 [file ijerph-19-06195-s001.zip › ijerph-1688500-supplementary.pdf]

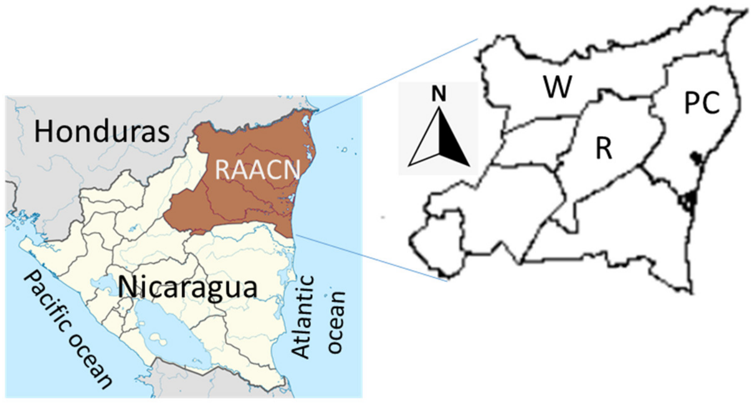

Figure S1. Geographic location of Nicaragua and Geographic location of Nicaragua and most affected municipalities by malaria transmission.

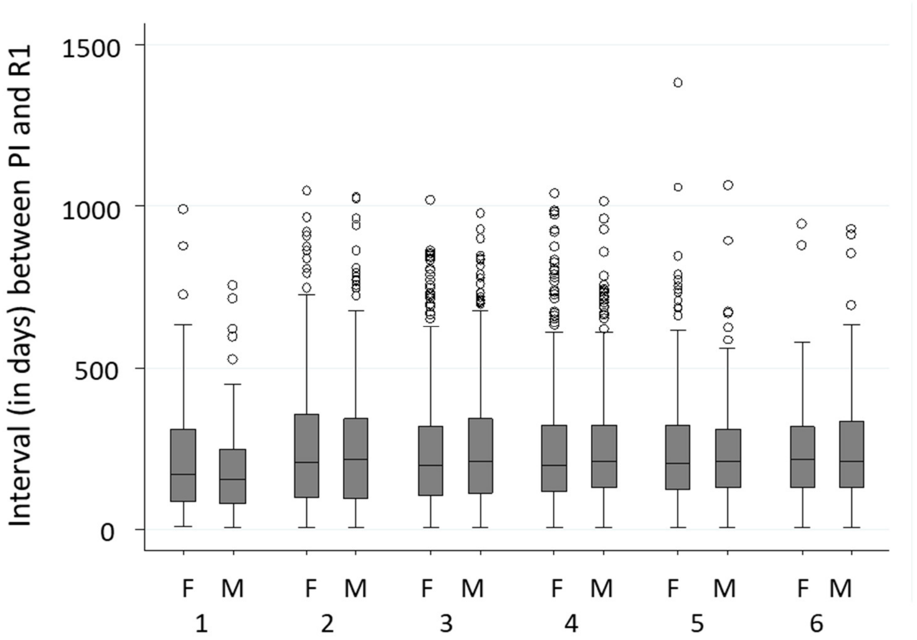

Figure S2. The time interval (≥25 days) between the primary case (PI) and first recurrent *P. vivax* case (R1) in Nicaragua from 2013–2018, depicted by gender and age group. Age groups: 1, 0–5 (n = 101); 2, 6–15 (n = 371); 3, 16–25 (n = 470); 4, 26–40 (n = 363); 5, 41–55 (n = 128); 6, >55 (n = 81). There was association between latency of R1 and patient age for women (Wilcoxon: $\chi^2(5) = 11.57$; $p = 0.041$). If the time interval was circumscribed to 25–450 days, no significance was detected ($\chi^2(5) = 8.48$; $p = 0.131$).

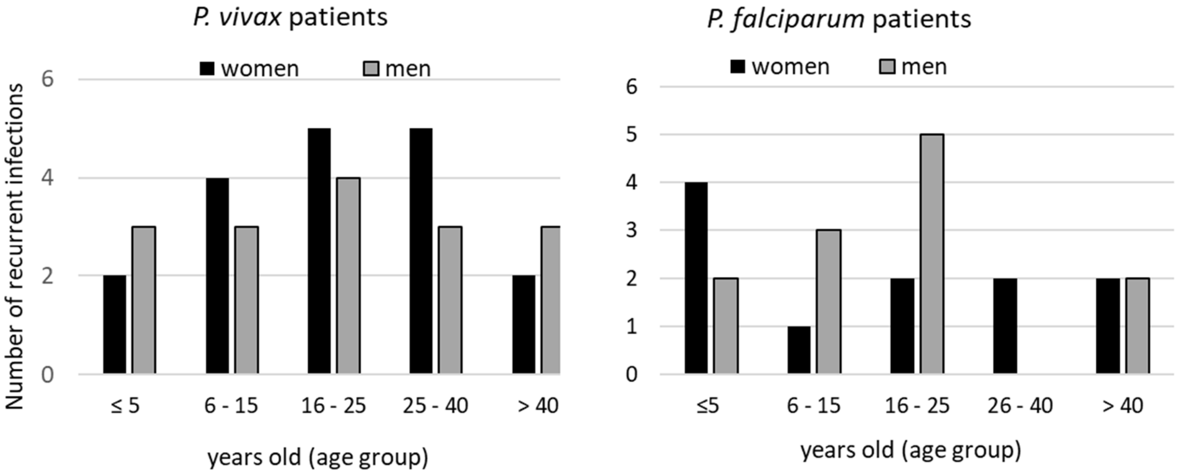

Figure S3. Distribution of patients with short-latency recurrent *P. vivax* and *P. falciparum* infections by age and gender. Data are for first reccurent *P. vivax* infections with a latency of 7–24 days (Mann-Whitney: z = −0.462, p = 0.643) and recurrent *P. falciparum* infections with a latency period of 7–60 days after the primary infection (z = −0.307, p = 0.758).

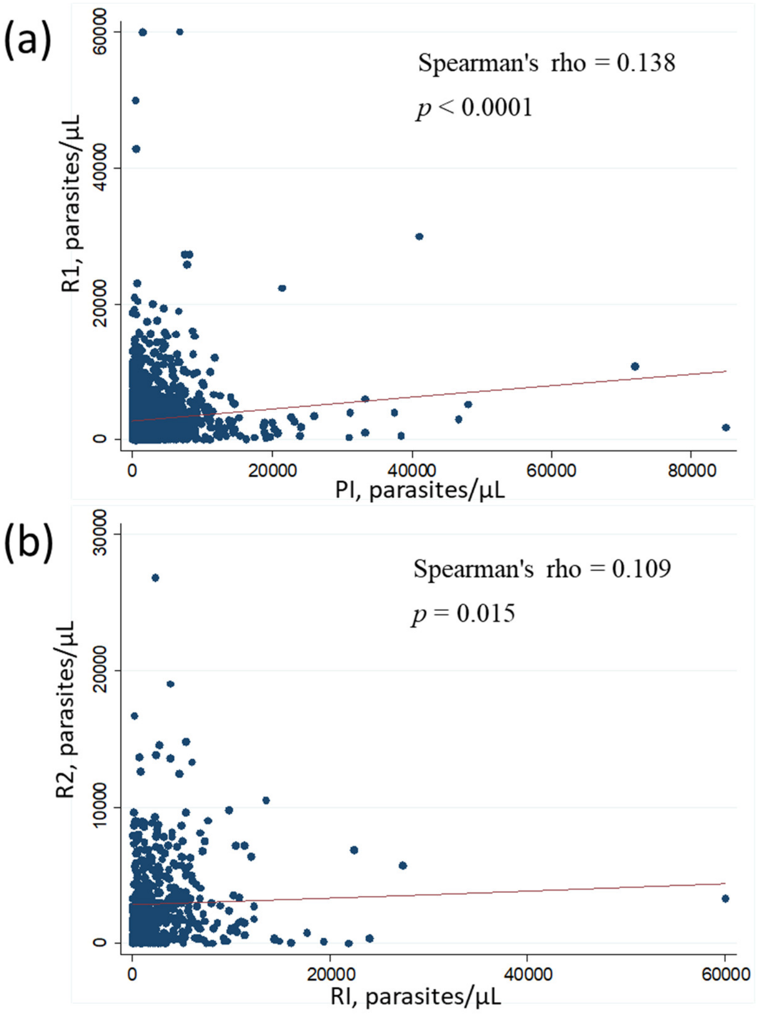

Figure S4. Correlation of parasitemia in consecutive *P. vivax* infections in Puerto Cabezas, Nicaragua from 2017–2018. PI, primary infection; R, recurrent infection; (**a**) PI/R1, $n = 1784$; (**b**) R1/R2, $n = 495$.

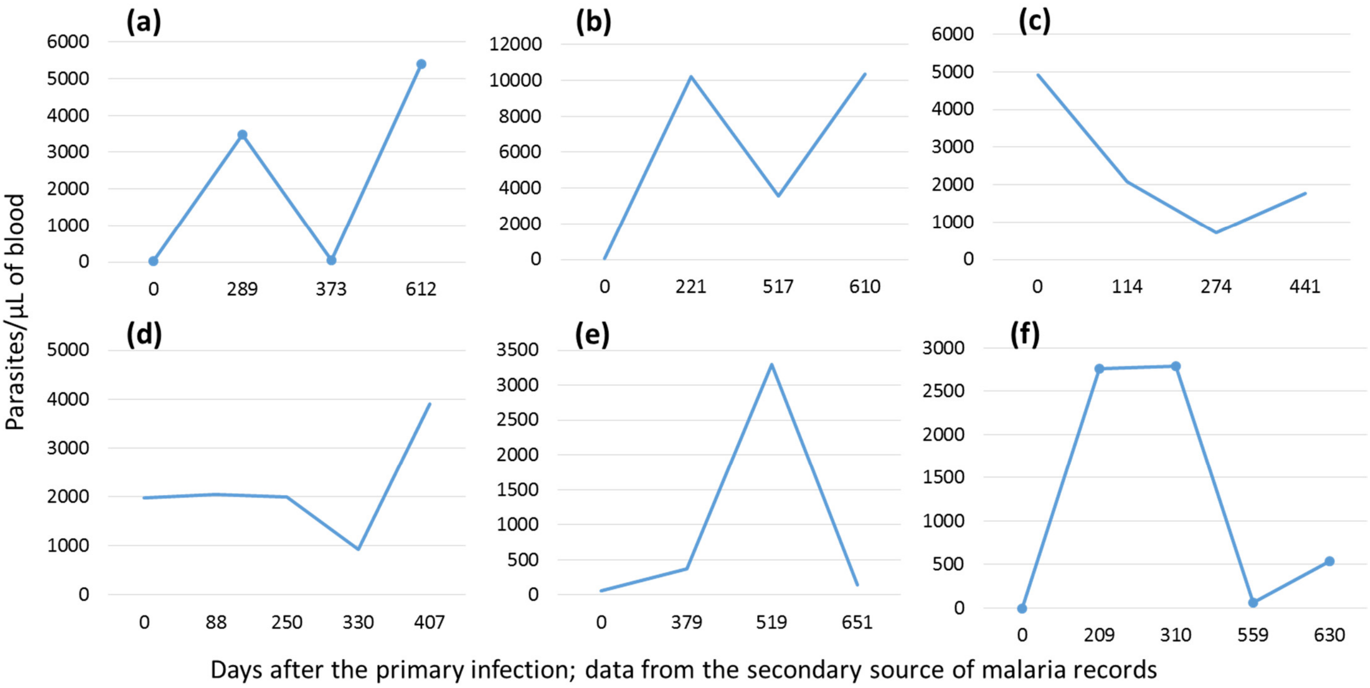

Figure S5. Variation of parasite density in primary and recurrent infections at a patient level. Based on data from Puerto Cabezas, Nicaragua (2017–2018), parasitemia is portrayed in 6 patients (**a**–**f**) with 3 recurrent episodes after the primary blood infection. Day 0, first diagnosed *P. vivax* infection.

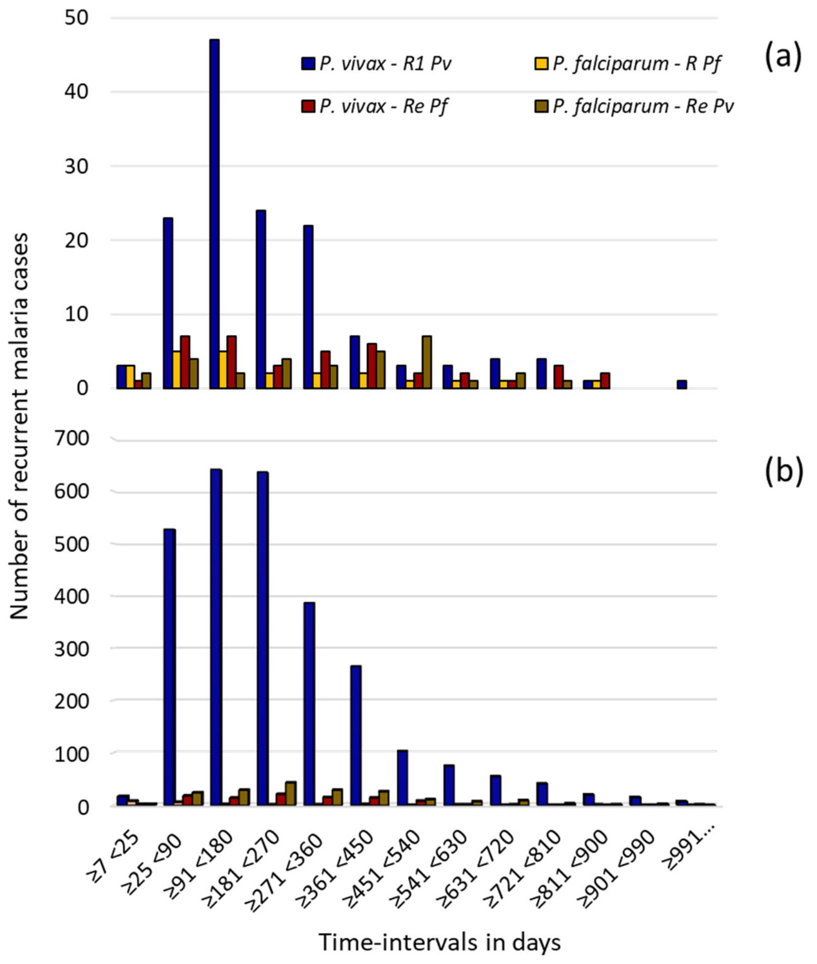

Figure S6. Comparison of *P. vivax* and *P. falciparum* recurrent infections and re-infections in two municipalities of RACCN, Nicaragua, 2013–2018. The frequency of homologous and heterogeneous infections (recurrence and reinfection, respectively) is categorized according to the interval of time between the primary infection and the first recurrent infection (R1) for Rosita (**a**) and Puerto Cabezas (**b**). R1 *Pv*, first recurrent *P. vivax* infection; R *Pf*, recurrent *P. falciparum* infection; Re, reinfection (two consecutive infections by heterologous malaria species).
